# DT-13 Ameliorates TNF-α-Induced Vascular Endothelial Hyperpermeability *via* Non-Muscle Myosin IIA and the Src/PI3K/Akt Signaling Pathway

**DOI:** 10.3389/fimmu.2017.00925

**Published:** 2017-08-14

**Authors:** Yuanyuan Zhang, Yuwei Han, Yazheng Zhao, Yanni Lv, Yang Hu, Yisha Tan, Xueyuan Bi, Boyang Yu, Junping Kou

**Affiliations:** ^1^State Key Laboratory of Natural Products, Jiangsu Key Laboratory of TCM Evaluation and Translational Research, Department of Complex Prescription of TCM, China Pharmaceutical University, Nanjing, China

**Keywords:** DT-13, vascular endothelial hyperpermeability, tight junctions, Src/PI3K/Akt, non-muscle myosin IIA

## Abstract

DT-13(25(R,S)-ruscogenin-1*-O-*[β-d-glucopyranosyl-(1→2)][β-d-xylopyranosyl-(1→3)]-β-d-fucopyranoside) has been identified as an important factor in TNF-α-induced vascular inflammation. However, the effect of DT-13 on TNF-α-induced endothelial permeability and the potential molecular mechanisms remain unclear. Hence, this study was undertaken to elucidate the protective effect of DT-13 on TNF-α-induced endothelial permeability and the underlying mechanisms *in vivo* and *in vitro*. The *in vivo* results showed that DT-13 could ameliorate endothelial permeability in mustard oil-induced plasma leakage in the skin and modulate ZO-1 organization. In addition, the *in vitro* results showed that pretreatment with DT-13 could increase the transendothelial electrical resistance value and decrease the sodium fluorescein permeability coefficient. Moreover, DT-13 altered the mRNA and protein levels of ZO-1 as determined by real-time PCR, Western blotting, and immunofluorescence analyses. DT-13 treatment decreased the phosphorylations of Src, PI3K, and Akt in TNF-α-treated human umbilical vein endothelial cells (HUVECs). Further analyses with PP2 (10 µM, inhibitor of Src) indicated that DT-13 modulated endothelial permeability in TNF-α-induced HUVECs in an Src-dependent manner. LY294002 (10 µM, PI3K inhibitor) also had the same effect on DT-13 but did not affect phosphorylation of Src. Following decreased expression of non-muscle myosin IIA (NMIIA), the effect of DT-13 on the phosphorylations of Src, PI3K, and Akt was abolished. This study provides pharmacological evidence showing that DT-13 significantly ameliorated the TNF-α-induced vascular endothelial hyperpermeability through modulation of the Src/PI3K/Akt pathway and NMIIA, which play an important role in this process.

## Introduction

Alterations in endothelial barrier function play an important role in the pathogenesis of many disease conditions, including immunology diseases, sepsis, inflammation, wound healing, edema, acute lung injury (ALI), stroke, and cancer (1–3). The integrity of the vascular endothelial cell layer is maintained by intercellular complexes composed of tight junctions (TJs), adherens junctions, and gap junctions (4). TJs, the most luminal component of the apical junctional complex, form a barrier that limits paracellular movement of water, ions, and macromolecules (5). Recently, TNF-α-induced barrier dysfunction has been shown to be a critical contributor to human disease. TNF-α-induced inflammatory changes to endothelial cells include protein synthesis-independent changes in cell permeability and cell motility, leading to localized inflammatory responses, vascular leakage, and edema formation. TNF-α-induced permeability underlies the pathobiology of several disorders (6, 7). Therefore, it is important to investigate the ability of drugs used to treat various diseases to protect endothelial cell permeability.

Different proteins have been shown to be located in TJs, including ZO-1, ZO-2, ZO-3, occludin, and claudins (8). TJs prevent diffusion of molecules between adjacent endothelial cells and maintain endothelial tightness. ZO-1 was the first TJ protein described, and it is critical for junction assembly and permeability. In the absence of ZO-1, cells fail to form TJs (9, 10). The ZO-1 protein not only combines with cytoplasmic and transmembrane proteins but also interacts with ZO-2 or ZO-3 to form a complex in the cell that principally acts as a bridge. The cell membrane protein occludin and the intracellular skeleton component actin interact to form a stable structural system, and the other proteins are closely involved in this structure (11, 12). In many cases, ZO-1 dysfunction or distribution changes destroy the integrity of the TJs between cells, causing gap increases, which result in increased vascular permeability. Thus, ZO-1 has been used as a marker of barrier integrity and permeability (13). ZO-1 knockout in Eph4 cells resulted in TJ damage (9).

Src family kinases are a class of non-receptor tyrosine kinases involved in the modulation of cell survival, proliferation, differentiation, and migration. In some cells, Src is a critical upstream regulator of steroid-stimulated membrane signal transduction pathways (14). Knockdown (KD) of Src accelerates oxidative stress-induced assembly of TJs and restoration of the barrier function (15). In addition, Src promotes PI3K *via* the Src association with the p85 subunit of PI3K (16). The class I PI3K and the serine/threonine-specific protein kinase Akt signaling pathway (PI3K/AKT) is one of the most important pathways involved in inflammatory responses (17). The increase in endothelial permeability and ZO-1 alteration induced by pro-inflammatory cytokines may be related to the PI3K/Akt pathway (18).

Non-muscle myosin II consists of a group of molecular motors, including three paralogs (IIA, IIB, and IIC), which are involved in a wide variety of cellular processes, including cytokinesis, proliferation, adhesion, migration, and control of cell morphology (19–21). Non-muscle myosin IIA (NMIIA) plays an important role in inflammation and could be a target for inflammatory disease therapy (22). In inflammatory bowel disease patients, steady-state levels of atypical PKC in the active conformation decrease with inflammation, while apical expression of IIA increases with inflammation, and there is a negative correlation between these signals. Myosin and actin form a dense ring that encircles the cell at the level of the adherens junction and TJ. Activation of actomyosin contraction, as assessed by phosphorylation of myosin II regulatory light chain (MLC), has been implicated in TJ regulation (23, 24). Notably, NMIIA plays in important role in modulating transcription factor expression and activity by interacting with TNFR2 and modulating the Akt/GSK3-NF-κB signaling pathways to inhibit thrombosis (25). Therefore, NMIIA may be an important target in inflammation treatment.

Steroidal saponin DT-13 (25(R,S)-ruscogenin-1-*O*-[β-d-glucopyranosyl-(1→2)][β-d-xylopyranosyl-(1→3)]-β-d-fucopyranoside) (Figure S1A in Supplementary Material), one of the active compounds isolated from Liriope *muscari*, has been shown to have various biological properties, including anti-inflammatory, cardioprotective, and antitumor activities (26–28). Recently, it was reported that DT-13 inhibited the phosphorylation of Src (Tyr416) in TNF-α-induced human umbilical vein endothelial cells (HUVECs) and could be safely used as a potential drug for vascular inflammation (29). DT-13 attenuated human lung cancer metastasis by regulating NMIIA activity under hypoxic conditions (30–32). However, it is not clear whether DT-13 plays a role in ameliorating vascular permeability.

Thus, in the present study, we showed that TNF-α significantly enhanced permeability in endothelial cells *via* a signaling pathway involving Src and PI3K/Akt, which led to the disruption of TJ proteins, such as ZO-1, and barrier dysfunction in the endothelial cells. These findings provide novel insight into the effect of DT-13 on endothelial barrier function, which is critical in vascular hyperpermeability-associated diseases. NMIIA may play an important role in the DT-13-mediated protection of against endothelial cell dysfunction.

## Materials and Methods

### Extraction and Isolation of DT-13

DT-13 was isolated as previously described and identified as (25(R,S)-ruscogenin-1*-O-*[β-d-glucopyranosyl-(1→2)][β-d-xylopyranosyl-(1→3)]-β-d-fucopyranoside) by comparison of its physical data (1H NMR, 13C NMR, MS) with published values. The purity of DT-13 was shown to be 98.5% using HPLC–ELSD assays as previously reported (33).

### Cell Culture

Human umbilical vein endothelial cells were purchased from the Shanghai Institute of Cell Biology, Chinese Academy of Sciences. HUVECs were grown in RPMI 1640 medium (Invitrogen Life Technologies, Carlsbad, CA, USA) supplemented with 10% heat-inactivated fetal bovine serum (FBS, ScienCell, CA, USA), 100 U/mL penicillin, 100 µg/mL streptomycin, and 2.0 g/L sodium bicarbonate. Cells were maintained at 37°C with 95% humidity and 5% CO_2_.

### Vascular Permeability Assay

Ten-week-old male C57BL/6 mice were purchased from Yangzhou University (Yangzhou, China, certificate NO. SCXK 2014-0004). Mice were housed in microisolator cages in a pathogen-free facility. Evans blue dye (30 mg/kg) in 100 µL PBS (Evans blue, Sigma, USA) was injected into the tail vein. After 1 min, mustard oil (Sigma, USA) diluted to 5% in sunflower seed oil was applied to the dorsal and ventral surfaces of the ear as described in previous studies (34). This process was repeated 15 min later. Then, photographs were taken 30 min after injection of Evans blue dye. After the mice were euthanized, the ears were removed, blotted dry, and weighed. The Evans blue dye was extracted from the ears with 1 mL of formamide overnight at 55°C, and the OD value at 600 nm was determined. All animal experimental procedures and welfare were in accordance to National Institutes of Health Guide for the Care and Use of Laboratory Animals, and the protocols used were approved by the Animal Ethics Committee of China Pharmaceutical University, China Pharmaceutical University, Nanjing, China.

### Animals and Experimental Design

After an initial acclimation period, the mice were randomly divided into four groups, with six mice per group. Mice were pretreated with DT-13 by intragastric administration with 4.0 mg/kg for 1 h. After 1 h, the mice were administered TNF-α by intraperitoneal injection with 100 µg/kg for 4 h. Mice received saline (0.9%), DT-13 or dexamethasone (Dex) for the same period. At the end of the experimental period, all mice were euthanized 2 h after the last TNF-α injection. Blood samples were collected, and serum was frozen at −80°C for the ELISA analysis.

### Immunofluorescence Staining

The immunofluorescence staining of ZO-1 *in vivo* was determined as described previously with a few modifications (35). Aortas from the mice were rapidly excised under general anesthesia, carefully trimmed to remove fat and connective tissue and washed twice by ice-cold PBS. Then the aortas were opened longitudinally to expose the endothelium and pinned onto 4% agar. HUVECs were cultured to confluence on glass cover slips in complete media containing 10% FBS and maintained for 7 days. Cells were then stimulated with TNF-α (10 ng/mL) for 4 h with or without prior treatment with DT-13 (1 µM) for 1 h. The aortas or cells were washed with PBS and fixed in 4% formaldehyde in PBS (v/v) for 30 min at room temperature, and permeabilized in 0.1% Triton X-100 in 5% bovine serum albumin (BSA, diluted in PBS) for 30 min at room temperature. The aortas or cells were then blocked with 5% BSA for 1 h at room temperature. Then, they were incubated with rabbit anti-ZO-1 polyclonal antibody overnight at 4°C and washed with PBS three times followed by incubation with donkey antirabbit IgG 488-conjugated secondary antibody for 1 h. All samples were assessed using a fluorescence microscope (LSM700, Zeiss, Germany).

### Transendothelial Electrical Resistance (TEER) Assays and Sodium Fluorescein (Na-F) Assays

Human umbilical vein endothelial cells were seeded on transwell inserts (0.4 µM pore, 6.5 mm diameter, Millipore, USA) for 7 days. The TEER of the monolayer was also measured daily with a Millicell-ERS voltohmeter (Millipore, USA). Resistance values of multiple transwell inserts of an experimental group were measured sequentially, and the mean was expressed in the common unit (Ω·cm^2^) after subtraction of the value of a blank cell-free filter. The TEER of the monolayers was recorded when a stable resistance reading was achieved with triplicate measurements that were taken for each transwell. DT-13 (0.01–1 µM) or Dex (1 µM) was added to the upper chamber for 1 h, and 10 ng/mL TNF-α (Bioworld, USA) was added for 4 h. Paracellular permeability was assessed by the addition of Krebs–Ringer buffer (118 mM NaCl, 4.7 mM KCl, 1.3 mM CaCl_2_, 1.2 mM MgCl_2_, 1.0 mM NaH_2_PO_4_, 25 mM NaHCO_3_, and 11 mM d-Glucose, pH 7.4) containing 100 µg/mL Na-F to the top chamber. The fluorescence was measured after 30 min at 37°C. The Na-F concentration was determined using a fluorescence multiwall plate reader [Ex (λ) 485 nm; Em (λ) 530 nm; Thermo].

### Transmission Electron Microscopy (TEM)

For direct examination of the ultrastructural characteristics of TJs, HUVECs were prepared using standard techniques and examined under a TEM (JEM1230, JEOL). Briefly, after fixation for 2 h with cold 2.5% glutaraldehyde and after several washes in 0.1 M PBS, the cells were gently scraped from the glass slides and pelleted by centrifugation. Cells were then post-fixed with 1% osmium tetroxide for 1 h at 4°C and stained en bloc with 2% uranyl acetate for an additional 1 h. After three more washes in double-distilled water, the samples were dehydrated in a series of acetone solutions and embedded in Epon 812 using a standard procedure. Random fields taken from individual endothelial cells samples were photographed at ×30,000.

### Reverse Transcription Quantitative Polymerase Chain Reaction (RT-PCR)

Human umbilical vein endothelial cells were seeded overnight at 3.0 × 10^5^ cells/dish. The cells were treated with or without DT-13 at a concentration of 1 µM for 1 h before exposure to TNF-α for 4 h. After cell stimulation, total RNA (four samples from each treatment group) was extracted using TRIzol reagent following the manufacturer’s protocol (Sunshine Biotechnology, Nanjing, China). Reverse transcription was performed using a First Strand cDNA synthesis kit (Vazyme, China), and qPCR was performed using SYBR Green Supermix (Bio-Rad, USA). The primer sequences used in RT-PCR were as follows: human 18 S, 5′-CAGCCACCCGAGATTGAGCA-3′ and 5′-TAGTAGCGACGGGCGGTGTG-3′; ZO-1, 5′-GCCTAATCTGACCTATGAAC-3′ and 5′-GGACTCGTATCTGTATGTG-3′. The amplification conditions were one cycle of 95°C for 30 s followed by 39 cycles of 95°C for 5 s, and 58°C for 15 s. mRNA expression was normalized to the level of 18 S expression. Changes in mRNA expression were calculated according to the 2^−ΔΔCT^ method, where ΔCT = CT_target gene_ − CT_18s_, and ΔΔCT = CT_treatment_ − ΔCT_control_, and CT is the value for cycle threshold.

### Western Blotting

Human umbilical vein endothelial cells were handled with various concentrations (0.01, 0.1, and 1 µM) of DT-13 for 1 h following TNF-α (10 ng/mL) stimulation for 4 h. After the cells were washed with PBS, they were lysed using lysis buffer (containing 20 mM Tris, pH 7.5, 1% Triton X-100, 150 mM NaCl, sodium pyrophosphate, β-glycerophosphate, EDTA, Na_3_VO_4_, leupeptin) for 30 min on ice. Protein concentration was measured using a BCA protein assay kit. Equal amounts of protein (40 µg) were separated using SDS–PAGE and then transferred onto a PVDF membrane. The membrane was then blocked with 3% BSA for 1.5 h, followed by overnight incubation at 4°C in the primary antibody. After the membrane was washed, a HRP-conjugated secondary antibody was added and incubated for 1.5 h. The bands were detected by an ECL kit and quantified by Quantity One software.

### siRNA Transfection

Non-muscle myosin IIA siRNA and control non-specific siRNA were purchased from Biomics Biotechnologies Co. (Nantong, China). Transfection of cells with the siRNAs was performed using ExFect Transfection Reagent (Vazyme, China) according to the manufacturer’s instructions. Briefly, the endothelial cells were plated on a 60 mm dish at a concentration of 5 × 10^5^ cells/dish. After reaching 50% confluence, the cells were transfected with the siRNA for 48 h and treated with DT-13 (0.01, 0.1, 1 µM) for 1 h followed by a 4 h treated with TNF-α (10 ng/mL) at 37°C. The siNMIIA sequences were as follows: forward, 5′-GAGGCAAUGAUCACUGACUdTdT-3′; reverse, 5′-AGUCAGUGAUCAUUGCCUCdTdT-3′. For analysis of the efficiency of protein suppression, total cell lysates were resolved by SDS–PAGE for immunoblotting with antibodies against GAPDH (Kangchen, China) and NMIIA (CST).

### Pose Validation, Molecular Preparation, and Docking

The protein docking was determined as described previously with a few modifications (36). Considering different state and sequences of the myosin models, the binding cavity was found by assistant methods based on the literature data (37). The precise binding cavity was determined *via* CH3 probe by Q-sitefinder [SP5] and blind docking by Autodock3.05 [SP6]. The steps were as follows: DT-13 and homology model of NMMHC IIA were prepared using ligprep and protein preparation wizard in Maestro 8.0 [SP7] adding hydrogen atoms and desalting. The next step comprised the grid generation performed by box size (32 Å × 32 Å × 32 Å). In this step, a distinct area of the structure was defined, in which the docking was performed. The grid box was manually positioned to comprise the whole ligand-binding site. The docking step was performed using the generated grid files and the prepared ligands, with the advanced option set as 14 Å × 14 Å × 14 Å. The initial complex conformation was subjected to further molecular minimization by Gromacs3.3 on an IBM system X3400 server in the AMBER03 force field environment. Topology parameters were self-generated by the Dundee Prodrg2 server. The same steps described for optimizing protein alone were also used for bound complexes. Molecular graphics were inspected in molecular virtual viewer1.2.0 (Molegro) [SP8] and Discovery Studio.

### Statistical Analysis

GraphPad Prism software (Version 4.0, GraphPad Software, Inc., San Diego, CA, USA) was used to perform the data analysis. Data are expressed as the mean ± SD. Statistical significance between different groups was calculated by a one-way ANOVA where appropriate or a Student’s two-tailed *t*-test; *P*-values below 0.05 were considered statistically significant.

## Results

### Effect of DT-13 on Vascular Permeability and ZO-1 Organization *In Vivo*

**Figure 1:**
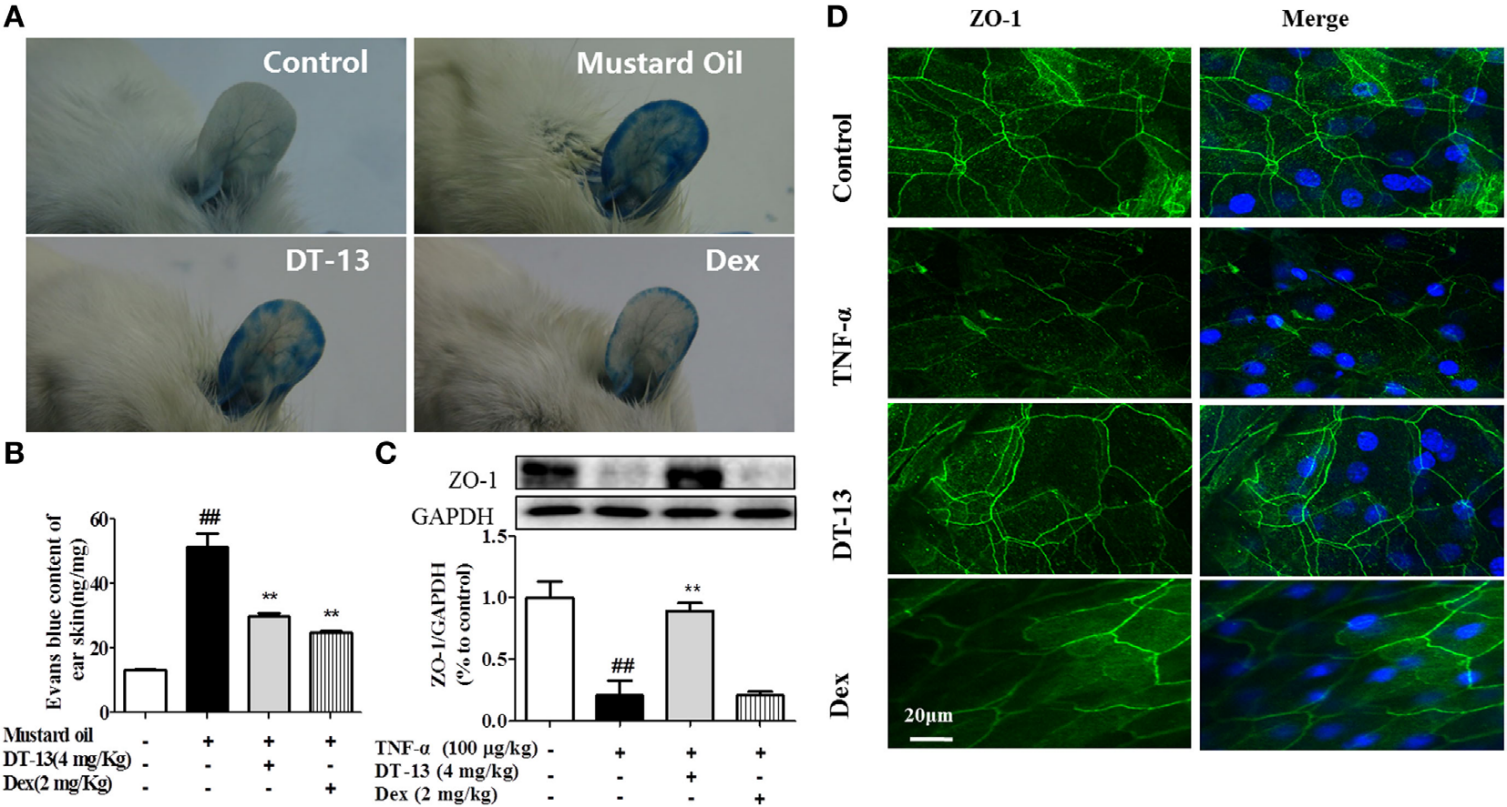
DT-13 prevented TNF-α-induced vascular hyperpermeability and ZO-1 disassembly *in vivo*. **(A)** Photographs of ears after treatment with mustard oil (30 min). **(B)** OD measurements of extravasated Evans blue in mouse ears 30 min after topical application of mustard oil. **(C)** The expression of ZO-1 in the mouse aorta was measured by Western blotting. **(D)** Immunostaining of ZO-1 in the mouse aorta endothelium. Bar = 20 μm. The ZO-1 in green and the DAPI in blue. The data represent the mean ± SD from three experiments. ^##^*P* < 0.01 vs. the control group; **P < 0.01 vs. the TNF-α group.

Dexamethasone was used in this part as a positive control to identify the successful construction of model *in vivo*. The response of vessels to mustard oil, an inflammatory agent that induces plasma leakage and inflammation in the skin, was examined. After injection of Evans blue dye and application of mustard oil, the ears of control mice became moderately blue. The ears of TNF-α-stimulated mice became completely blue, particularly at the periphery. In contrast, the ears of mice treated with DT-13 remained pale (Figure 1A). The group treated with Dex remained pale. Evans blue OD analysis revealed that leakage increased fourfold in TNF-α-stimulated mice. However, leakage did not increase significantly in the ear skin of mice treated with DT-13 and Dex (Figure 1B). As shown in Figure 1C, mice were pretreated with DT-13 (4.0 mg/kg) or Dex (2.0 mg/kg) for 1 h and then administered TNF-α (100 µg/kg) for 4 h. The protein expression of ZO-1 was significantly increased compared with that of the model group. Immunostaining of ZO-1 in the mouse aorta endothelium showed that TNF-α abolished the endothelial junctional protein at the cell–cell contact zones between adjacent cells. DT-13 (4.0 mg/kg) inhibited ZO-1 disassembly (Figure 1D). Notably, DT-13 was as potent as the glucocorticoid Dex.

### Effect of DT-13 on Endothelial Monolayer Permeability and Ultrastructural Characteristics of TJs *In Vitro*

**Figure 2:**
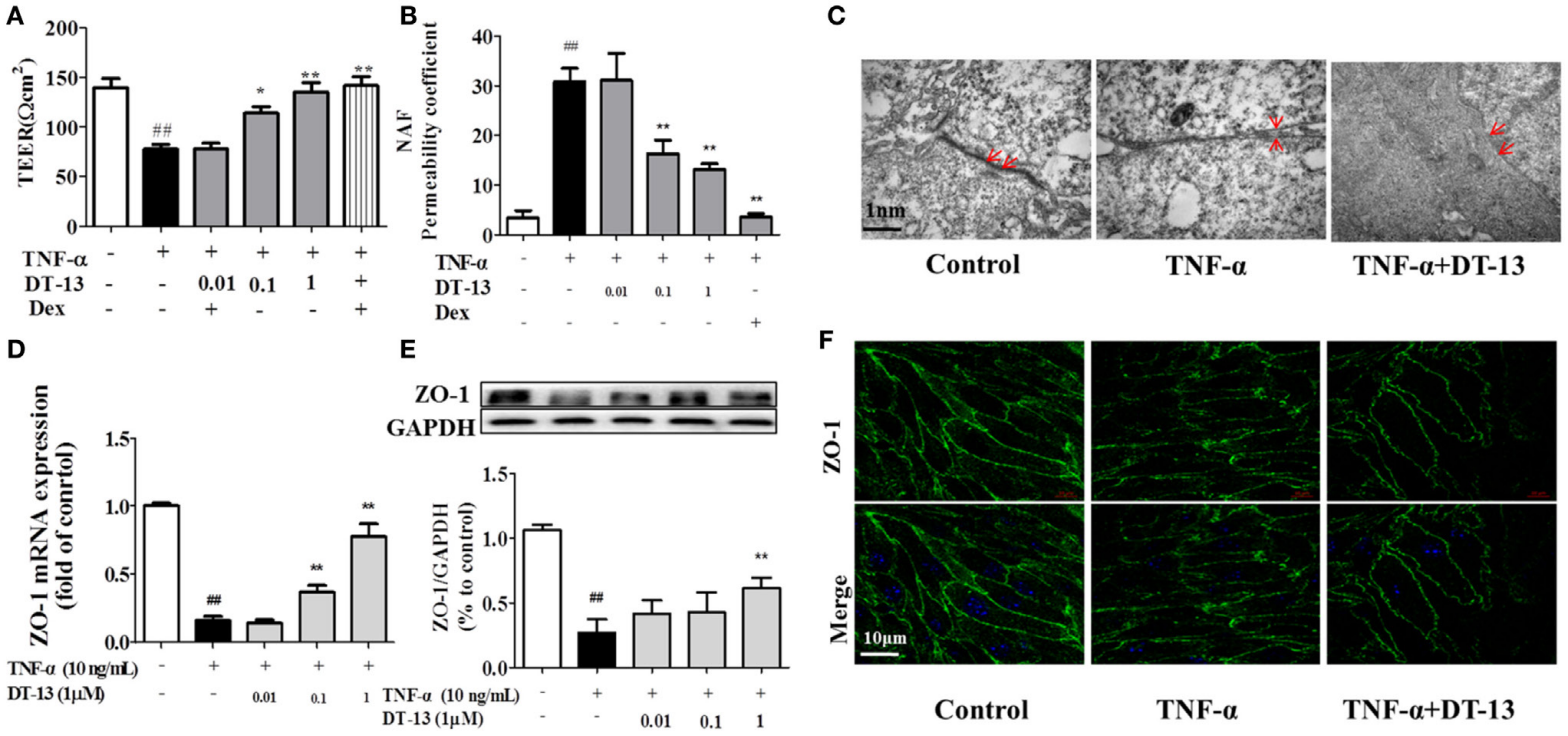
DT-13 ameliorated TNF-α-induced endothelial hyperpermeability. **(A)** EC permeability was measured using a Millicell-ERS voltohmeter. Human umbilical vein endothelial cells (HUVECs) were pretreated with various concentrations of DT-13 (0.01, 0.1, and 1 µM) or dexamethasone (1 µM) for 1 h followed by TNF-α (10 ng/mL) stimulation for 4 h. **(B)** The transendothelial permeability was assessed using the paracellular transport marker (sodium fluorescein) permeability coefficient, which was measured using a fluorescence multiwall plate reader [Ex (λ) 485 nm; Em (λ) 530 nm]. **(C)** Ultrastructural characteristics of tight junctions (TJs) (magnification ×50,000). TJs were intact in the group control; TJs were disrupted in the TNF-α-induced group; TJs were not disrupted in HUVECs pretreated with DT-13 (1 µM). **(D)** The mRNA level of ZO-1 was determined by real-time PCR. High-dose and moderate-dose DT-13 groups showed increases in the mRNA level of ZO-1 following TNF-α treatment. **(E)** Western blotting analysis revealed the effects of high-dose DT-13 on the protein expression of ZO-1, whereas the moderate-dose DT-13 group exhibited no significant change. **(F)** ZO-1 localization within the TJs of endothelial cells is altered by TNF-α, which was inhibited by DT-13. The ZO-1 in green and the DAPI in blue. Bar = 50 μm. The data represent the mean ± SD from three experiments. ^##^*P* < 0.01 vs. the control group; *P < 0.05, **P < 0.01 vs. the TNF-α group.

The effects of TNF-α or DT-13 on HUVEC monolayer permeability were examined by detecting the TEER of the EC monolayer and performing transwell Na-F assays. DT-13 at concentrations of 0.01, 0.1, and 1 µM was applied for 1 h, followed by TNF-α stimulation for 4 h. DT-13 or TNF-α had no effect on HUVEC viability (Figure S1B in Supplementary Material). TEER was reduced in the TNF-α group and DT-13 (1 µM) group by 42.3 and 0.3%, respectively (Figure 2A). TNF-α-induced barrier disruption resulted in a sharp decrease in TEER and an increase in the Na-F permeability coefficient. However, DT-13 enhanced TEER and decreased the Na-F permeability coefficient (Figure 2B). As a positive control, 1 µM Dex protected endothelial cell permeability. In the control group, the endothelial cells were tightly arranged. In contrast, large intercellular gaps were observed after TNF-α stimulation. However, the structure in the DT-13 group was undisrupted (Figure 2C).

Downregulation of the TJ protein ZO-1 may be critical for disruption of the endothelial barrier (13). This protein showed discrete localization in response to TNF-α treatment; however, ZO-1 in HUVECs treated with DT-13 was intact, in contrast to the group model. HUVECs were exposed to TNF-α and assessed for the expression of ZO-1 by RT-qPCR and Western blot analyses. As shown in Figures 2D,E, treatment of the HUVECs with TNF-α decreased the protein and mRNA expression of ZO-1, and this effect was significantly ameliorated in HUVECs pretreated with DT-13 at 1 µM. The ZO-1 protein maintains the structural integrity of TJs in endothelial cells. Using immunofluorescence, we identified the localization of ZO-1 at the endothelial cell contacts of confluent cells (Figure 2F).

### Effect of DT-13 on TNF-α-Induced Src/PI3-Kinase/Akt Pathway

**Figure 3:**
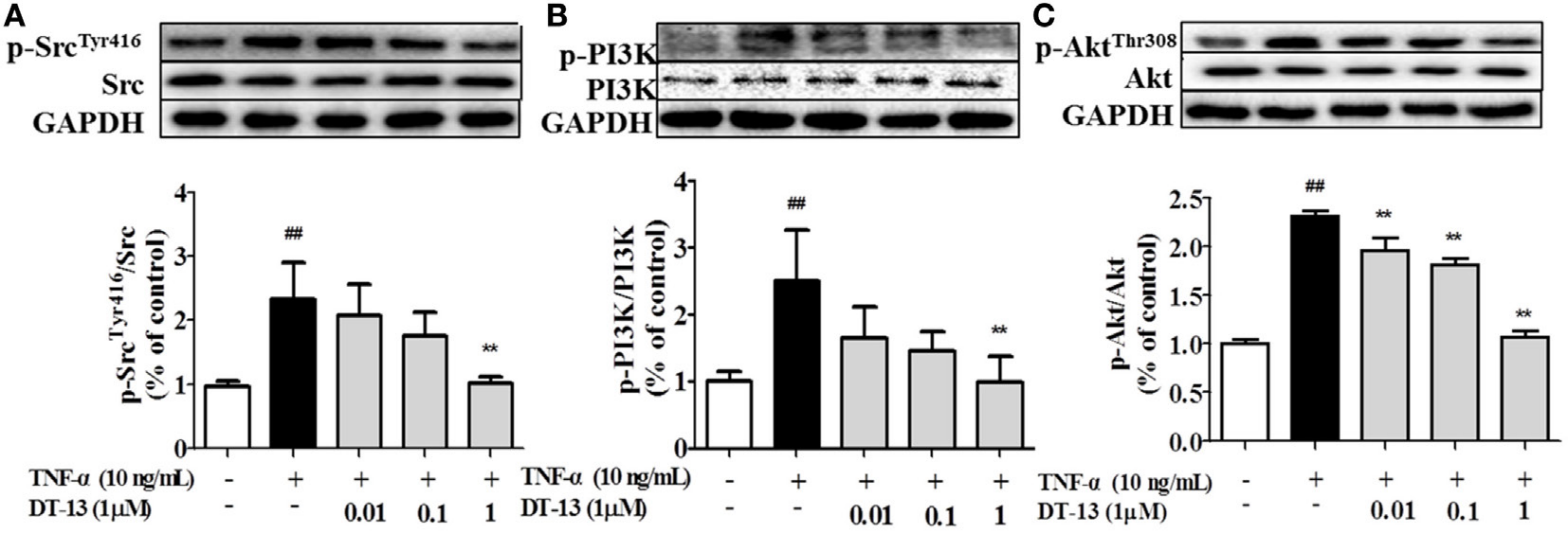
DT-13 modulated Src/PI3K/Akt pathway proteins in human umbilical vein endothelial cells (HUVECs). **(A)** Western blotting analyses of Src, **(B)** PI3K, and **(C)** Akt were performed after 4 h of 10 ng/mL TNF-α stimulation with or without various concentrations of DT-13 (0.01, 0.1, 1 µM) pretreatment for 1 h. The blots are representative images from three separate experiments. DT-13 decreased the expression of p-Src, p-PI3K, and p-Akt in HUVECs. The data represent the mean ± SD from three experiments. ^##^*P* < 0.01 vs. the control group; **P < 0.01 vs. the TNF-α group.

Pro-inflammatory cytokines increase the permeability and disassembly of ZO-1 *via* the PI3K/Akt pathway (38). Figure 3 shows that TNF-α increased Src, PI3K, and Akt phosphorylations in HUVECs. DT-13 dose-dependently blocked TNF-α-induced Src phosphorylation (Tyr416) and simultaneously abolished TNF-α-induced PI3K and Akt phosphorylation.

### DT-13 Attenuates TNF-α-Induced Disassembly Mediated by the Src/PI3-Kinase/Akt Pathway

**Figure 4:**
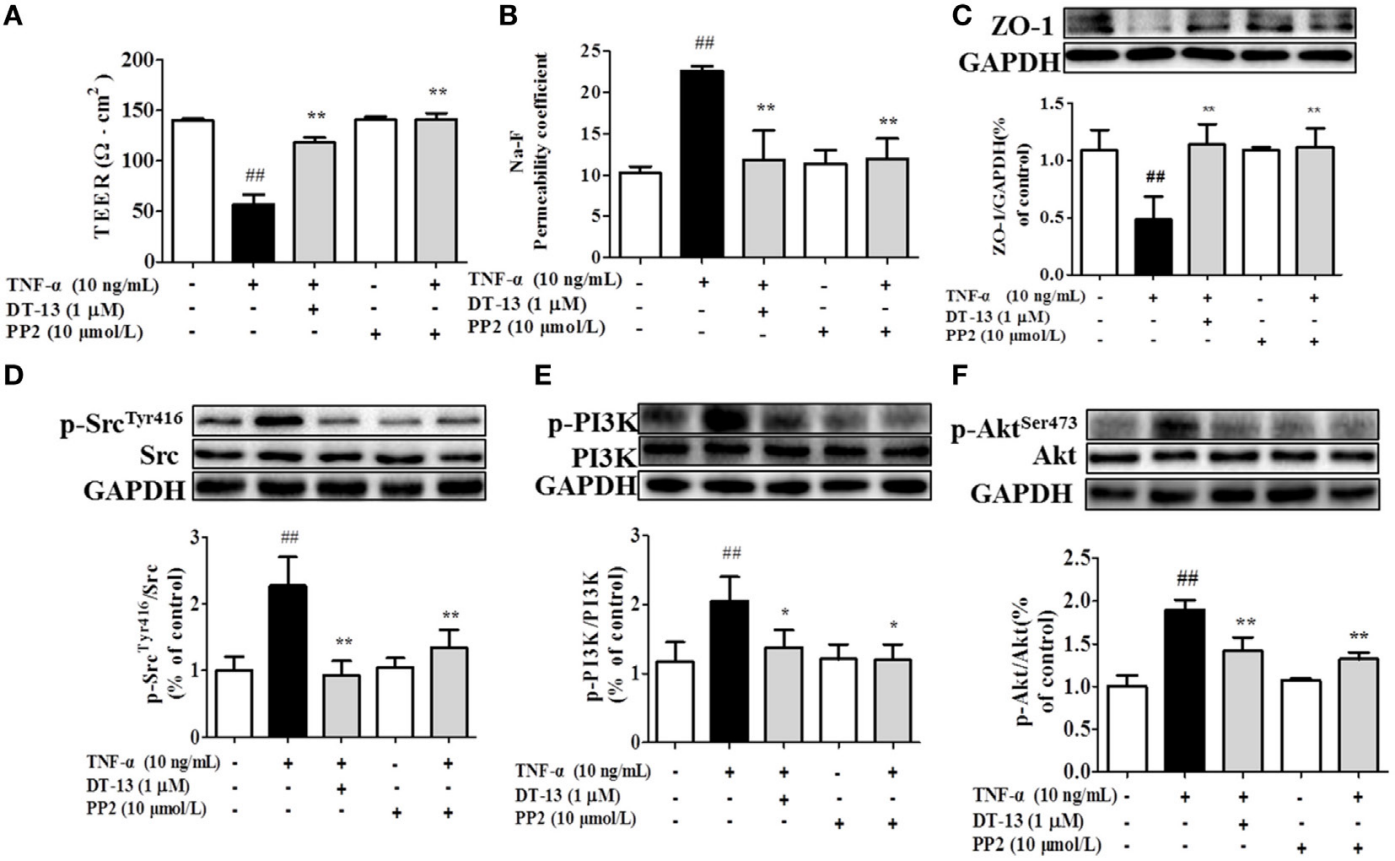
The Src pathway was related to DT-13 regulation of tight junction function induced by TNF-α. Permeability was assessed by transendothelial electrical resistance (TEER) and permeability of sodium fluorescein (Na-F) across the human umbilical vein endothelial cell (HUVEC) monolayer. **(A)** TEER was significantly elevated in HUVECs pretreated with 10 µM PP2. **(B)** Na-F permeability was significantly reduced in HUVECs pretreated with 10 µM PP2. Western blotting analysis for the effect of DT-13 on **(C)** ZO-1, **(D)** phosphorylation of Src, **(E)** PI3K, and **(F)** Akt in endothelial cells. The cells were pretreated with 10 µM PP2 for 1 h prior to addition of 1 µM DT-13 for 1 h and were then incubated with TNF-α for another 4 h for Western blot analysis. The data represent the mean ± SD from three experiments. ^##^*P* < 0.01 vs. the control group; *P < 0.05, **P < 0.01 vs. the TNF-α group.

**Figure 5:**
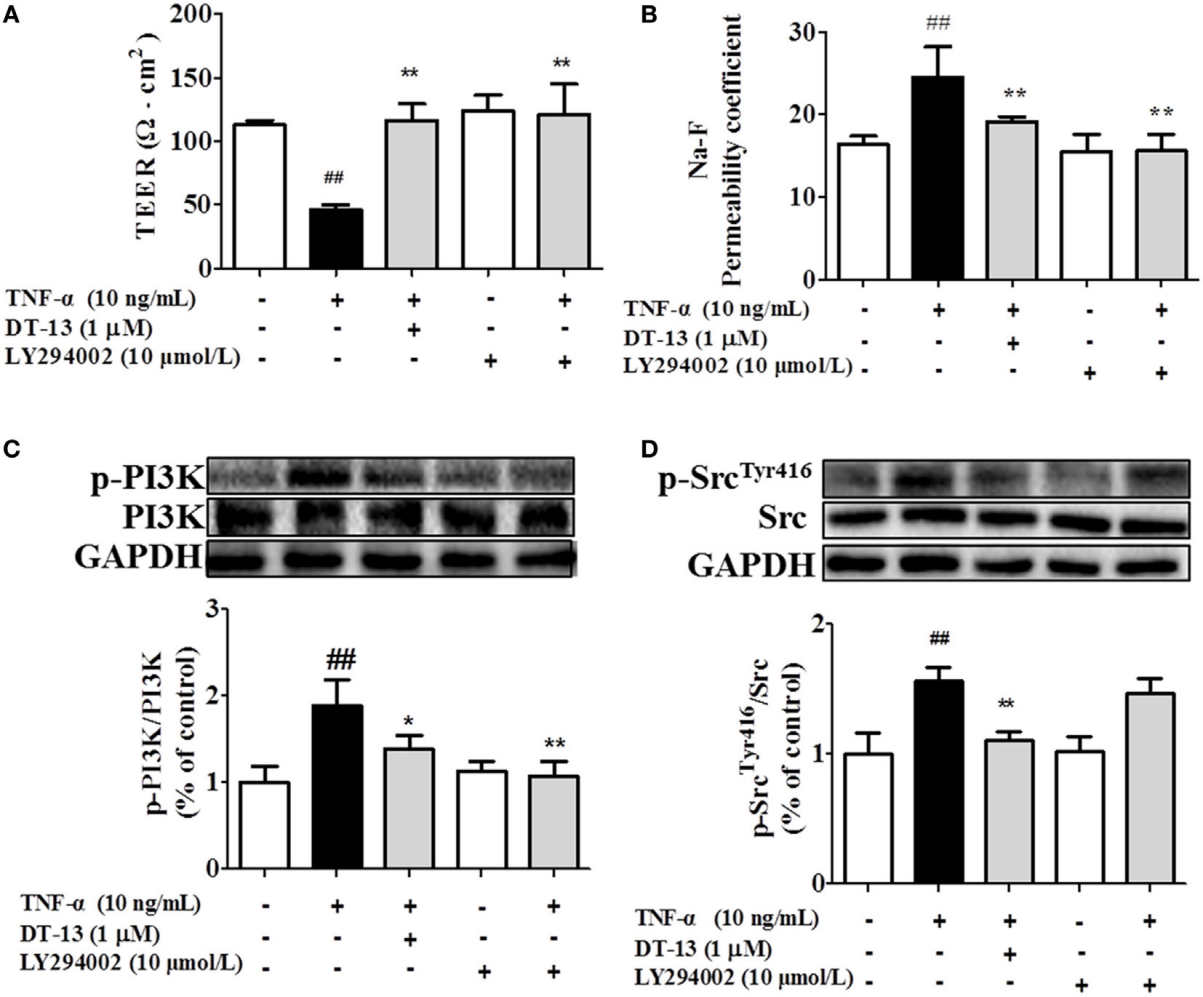
PI3K regulated TNF-α-induced permeability downstream of Src. Permeability was assessed by transendothelial electrical resistance (TEER) and permeability of sodium fluorescein (Na-F) across the human umbilical vein endothelial cell (HUVEC) monolayer. **(A)** TEER was significantly elevated in HUVECs pretreated with 10 µM LY294002. **(B)** Na-F permeability was significantly reduced in HUVECs pretreated with 10 µM LY294002. Western blotting analysis for the effect of DT-13 on **(C)** PI3K and **(D)** Src. Endothelial cells were pretreated with 10 µM LY294002 for 1 h before addition of 1 µM DT-13 for 1 h and then incubated with TNF-α for another 4 h for Western blot analysis. The data represent the mean ± SD from three experiments. ^##^*P* < 0.01 vs. the control group; *P < 0.05, **P < 0.01 vs. the TNF-α group.

Src family kinase activity was also shown to be important for TNF-α-mediated stimulation of PI3K/AKT activity because an Src inhibitor significantly prevented this. To determine the significance of Src, we carried out permeability studies. After HUVECs were pretreated with PP2 for 24 h, TEER values and the Na-F permeability coefficient were detected. TEER values in the TNF-α + PP2 groups were significantly increased compared with those in the TNF-α group (Figure 4A), and the Na-F permeability coefficient decreased (Figure 4B). Endothelial permeability is regulated in part by the endothelial cell-cell TJs, which are largely composed of ZO-1 (39). We hypothesized that DT-13 may decrease vascular permeability *via* an Src-induced disruption of ZO-1 localization to endothelial cell junctions. As expected, in control cells, ZO-1 was highly expressed (Figures 4C,D). Following treatment with 10 ng/mL TNF-α, lower levels of ZO-1 were found. However, pretreatment with the Src inhibitor PP2 (10 µM) for 1 h prior to TNF-α blocked the disassembly of ZO-1 (40). Inhibition of Src or DT-13 in HUVECs reduced the phosphorylations of Src, PI3K, and Akt. The cells were pretreated with the PI3K inhibitor LY294002 (10 µM) for 1 h prior to TNF-α, and TEER values and the Na-F permeability coefficient were detected (Figures 5A,B). TEER values in the TNF-α + LY294002 group were significantly increased compared with those in the TNF-α group. The Na-F permeability coefficient decreased. The expression of p-PI3K was decreased compared with that of the model group. However, in the same condition, the expression of p-Src was not significantly different from that of the model group (Figures 5C,D).

### NMIIA Plays an Important Role in DT-13-Attenuated TNF-α-Induced Endothelial Hyperpermeability and Related Pathways

**Figure 6:**
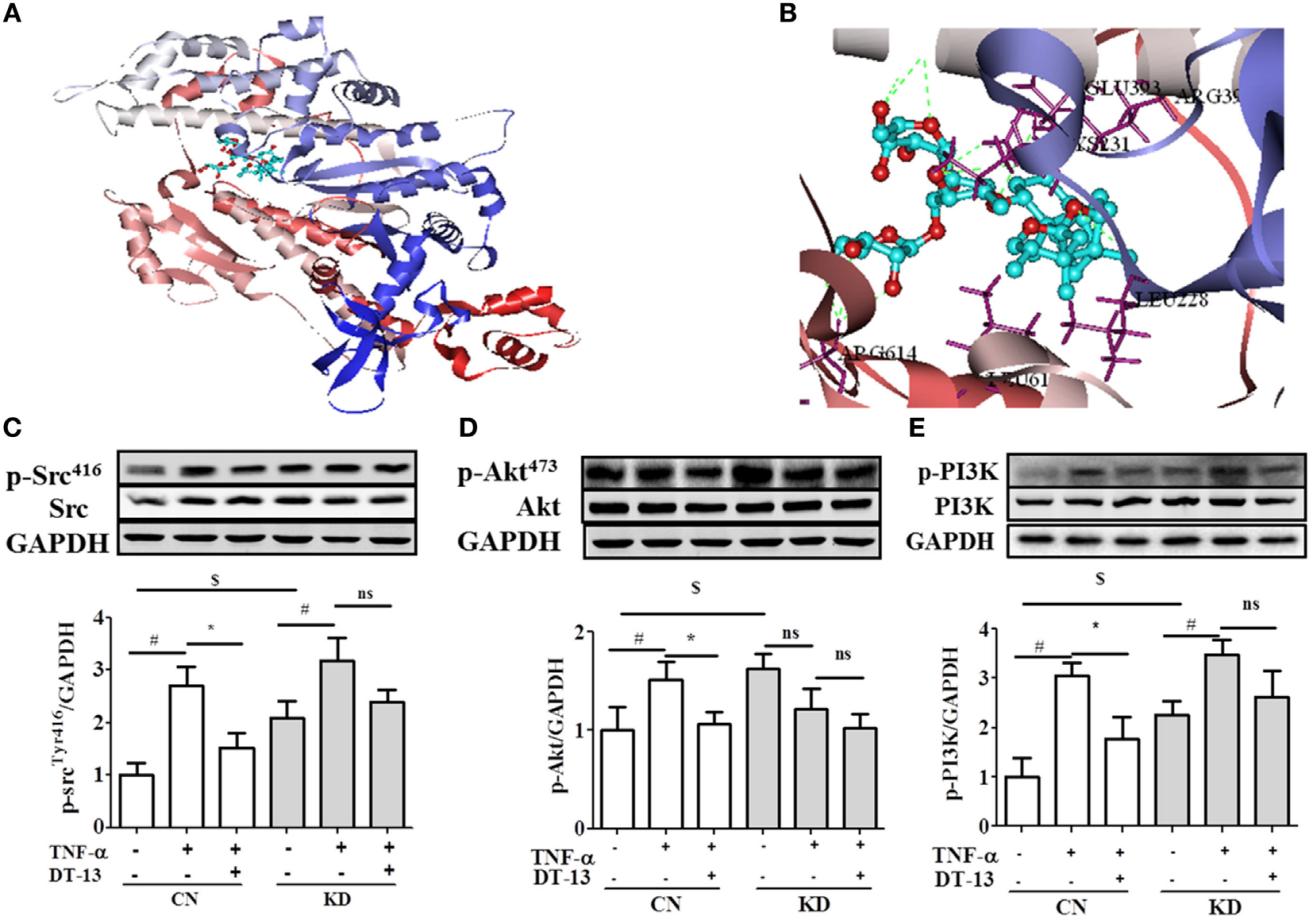
Non-muscle myosin IIA (NMIIA) plays an important role in DT-13-attenuated TNF-α-induced endothelial hyperpermeability and related pathways. **(A)** The complex of DT-13 and proteins of NMMHC IIA. **(B)** 5 Å residues of NMMHC IIA around DT-13. Western blotting analysis for the effect of DT-13 on **(C)** Src, **(D)** Akt, and **(E)** PI3K. Endothelial cells were pretreated with NMIIA siRNA for 43 h before addition of 1 µM DT-13 for 1 h and then incubated with TNF-α for another 4 h for Western blot analysis. The data represent the mean ± SD from three experiments. ^#^*P* < 0.01 vs. the control group; *P < 0.05, **P < 0.01 vs. the TNF-α group.

Computer aided methods were then used to construct a virtual model of NMMHC IIA by homology modeling and to illuminate the possible binding sites and binding mode of DT-13. Based on receptor structure combined with docking pharmacophore virtual screening method with G-score (−6.5), it was found that the DT-13 might locate into the cleft region similar to the inhibitor of NMMHC IIA and interacted with various binding sites including functional domains of ATPase, actin-binding or tail area of NMMHC IIA. As shown in Figure 6C, the basal expression of p-Src (Tyr416) increased in the NMIIA KD group (the expression of NMIIA was knocked down by the siRNA shown in Figure S1C in Supplementary Material) compared with that of the control HUVECs. These results indicated that NMIIA KD increase Src phosphorylation at Tyr416. However, the DT-13-mediated inhibition of Src Tyr416 was reduced in NMIIA KD HUVECs. The NMIIA KD abolished the effect of DT-13 on Src Tyr 416 phosphorylation. As shown in Figure 6D, the basal expression of p-Akt (473) increased in NMIIA KD HUVECs. DT-13 inhibited Akt phosphorylation by 30%. However, there was no response to TNF-α-induction, and DT-13 did not inhibit p-Akt (473) expression in NMIIA KD HUVECs. As shown in Figure 6E, the basal expression of p-PI3K increased in NMIIA KD HUVECs. DT-13 could inhibit the PI3K phosphorylation by 41%. PI3K phosphorylation was stimulated by TNF-α in NMIIA KD cells. However, DT-13 did not inhibit p-PI3K expression in NMIIA KD cells. These result indicated that NMIIA is essential for the DT-13-mediated effect on the PI3K/Akt pathway.

## Discussion

In this study, we demonstrated that DT-13, one of the major active compounds of *L. muscari*, suppressed the increase in TNF-α-stimulated endothelial cell permeability and the disassembly of ZO-1, possibly due to inhibition of the PI3K/Akt signaling pathway by modulating Src activation. These findings provide pharmacological evidence for the protective effect of DT-13 in treating cardiovascular disease and cancer, and the underlying mechanisms may be *via* modulation of TJs through the Src/PI3K/Akt signaling pathway. Moreover, NMIIA is essential for DT-13-mediated protection of endothelial cell permeability.

Vascular injury leads to endothelial injury and structural damage to TJs, resulting in systemic inflammatory response syndrome and sepsis (41). Endothelial hyperpermeability is a common characteristic of many diseases, including inflammation, trauma, sepsis, ischemia-reperfusion injury, diabetes, and atherosclerosis (42–45). In ALI, the endothelial cell barrier is weakened, leading to increased vascular permeability (46). As a crucial event in the progression of atherosclerosis, disruption of endothelial TJs accounts for the majority of the cardiovascular disease-related deaths (47). The blood–brain barrier (BBB) and the blood retinal barrier (BRB) are particularly rich in endothelial TJs. BBB/BRB disruption results in central nervous system diseases. Vascular leakage not only causes multiorgan dysfunction but also compromises the normal pharmacokinetics of therapeutic drugs (48). The inflammatory agent mustard oil is known to cause plasma leakage *via* increased numbers of leakage sites in the endothelium in mouse skin (49). In this study, mustard oil was used to induce hyperpermeability *in vivo*. TNF-α-induced vascular permeability is a critical contributor to the pathophysiology of vascular insufficiency (50). Pro-inflammatory mediators and neutrophils induced vascular hyperpermeability by Src signaling (51). Studies have showed that oxidants, VEGF, thrombin, neutrophils, and TNF-α increased Src activity in association with increased endothelial permeability (52, 53). TNF-α is a major pro-inflammatory cytokine that increases vascular permeability *via* disruption of intercellular junctions. Drugs can extravasate and accumulate inside the interstitial space; thus, their bioavailability and effectiveness are therefore reduced, and systemic toxicity can increase (54). In this report, we showed for the first time that DT-13 affects endothelial cell permeability induced by mustard oil *in vivo* and TNF-α *in vitro* (Figures 1 and 2). Therefore, we hypothesize that DT-13 may play a cardioprotective, antitumor, anti-inflammatory, and antistroke role *via* the effect on TJs (Figure 1).

ZO-1 is essential for the proper assembly of endothelial junction complexes that control endothelial junctional barrier integrity (55). TNF-α decreased the protein and mRNA content of the TJ protein ZO-1 and altered the endothelial cell permeability. The total content of the TJ protein ZO-1 under experimental conditions was determined by Western blotting and RT-PCR. Pretreatment with DT-13 increased TEER and up-regulated ZO-1. DT-13 was shown to enhance vascular TJ barrier integrity by up-regulation and rearrangement of ZO-1 (Figures 1 and 2).

Recent studies have elucidated the mechanisms by which Src family kinases regulate normal and pathological processes in vascular biology, including endothelial cell proliferation and permeability (56). There were reports indicated that Src tyrosine kinases promote inflammatory processes under various pathological conditions induced by TNF-α (57). Activation of PI3K appears to occur *via* phosphorylation of tyrosine residues in Src, which permits docking of PI3K to the plasma membrane and allows allosteric modifications that increase its catalytic activity (58). The Src inhibitor PP2 blocked the PI3K/Akt-mediated alteration in ZO-1 expression (59). Pretreatment with the PI3K inhibitor LY294002 blocked alteration in ZO-1 expression. Inhibition of Src in TNF-α-treated HUVECs decreased permeability and up-regulated expression of the endothelial cell TJ protein ZO-1, and this effect was mediated by inhibition of PI3K/Akt signaling (60). Our results indicated that DT-13 enhances vascular TJ barrier integrity through a mechanism involving Src and the PI3K/Akt pathway. Together, our data showed that DT-13 decreased endothelial cell permeability by enhancing endothelial cell TJ protein expression and that the Src/PI3K/Akt signal pathway is involved in this process (shown in Figures 3–5).

Myosin and actin form a dense ring that encircles the cell at the level of the adherens junction and TJ. The results of the docking-based virtual screening showed that NMIIA may be the combined protein of DT-13. Activation of actomyosin contraction, as assessed by phosphorylation of MLC, has been implicated in TJ regulation (23, 24). KD studies have shown that NMIIA activation is necessary for TJ response to diverse physiological and pathophysiological stimuli (61). Our data indicated that blocking NMIIA activation in HUVECs is sufficient to inhibit the effect of DT-13 on the Src/PI3K/Akt pathway. In this study, the expression of NMIIA was inhibited by siRNA, and the effect of DT-13 on the Src/PI3K/Akt signaling pathway was decreased. These results suggest that the regulatory effect of DT-13 on the Src/PI3K/Akt signaling pathway was closely related to NMIIA (shown in Figure 6). Thus, NMIIA may mediate DT-13 regulation of the Src/PI3K/Akt signaling pathway to improve endothelial hyperpermeability.

The major aim of the present study was to investigate the effect of DT-13 on TNF-α-induced endothelial hyperpermeability. Natural products have long been recognized as a key source for the development of novel therapeutic strategies to treat human disorders, including inflammatory diseases. Tongxinluo, a traditional Chinese medicinal compound, increased TJ protein expression after chronic hypoxia both *in vivo* and *in vitro* (62). He-Ying-Qing-Re Formula, a well-known traditional Chinese medicine, was shown to protect endothelial dysfunction *via* modulation of TJs (63). We demonstrated for the first time that DT-13 suppressed the increase in TNF-α-stimulated EC permeability and the disassembly of ZO-1, possibly due to inhibition of the PI3K/Akt signaling pathway by modulating Src activation. NMIIA may be the key mediator of DT-13-induced endothelial hyperpermeability amelioration. Moreover, we demonstrated that DT-13 could improve vascular barrier function. Thus, DT-13 could be developed as a drug to treat diseases related to vascular dysfunction, such as hypertension, stroke, and others.

Our results first confirmed that NMIIA has played an important role in the DT-13-mediated effect on the Src/PI3K/Akt pathway. As reported, saponin compounds from Chinese herbal could be the inhibitor of NM IIA based on combined pharmacophore- and docking-based virtual by computer calculation (36). However, further studies are needed to explore. First, the effect of DT-13 on the NM IIA ATPase activity needed to be investigated in future studies. Second, how the conditional NMIIA-KO in endothelial cells contributes in vascular barrier function requires further study. On the other hand, our results indicated that DT-13 enhances vascular TJ barrier integrity through a mechanism involving Src and the PI3K/Akt pathway as determined using the Src inhibitor PP2 and PI3K inhibitor LY294002. KD or knock out studies of Src and PI3K should be performed in the future.

Overall, our findings provide pharmacological evidence supporting the protective effect of DT-13 in treating cardiovascular disease and cancer. The underlying mechanisms are mediated *via* modulation of TJs through the Src/PI3K/Akt signaling pathway. More importantly, we demonstrated that NMIIA was essential in the process of DT-13-improved endothelial hyperpermeability. Our results provide new insights into the effects and mechanisms of DT-13 on endothelial hyperpermeability, which play an important role for the onset and progression of cardiovascular diseases, such as atherogenesis, cancer, and stroke. Therefore, DT-13 may be a potential candidate drug for immunology disease intervention.

## Ethics Statement

All animal welfare and experimental procedures were in accordance with National Institutes of Health Guide for the Care and Use of Laboratory Animals, and the protocols used were approved by the Animal Ethics Committee of China Pharmaceutical University, China Pharmaceutical University, Nanjing, China.

## Author Contributions

YZhang analyzed the data, wrote, discussed, and reviewed the manuscript. YHan wrote the manuscript and performed the experiments *in vivo* and intro. YZhao performed the experiments *in vivo* and intro. YL and YT performed the experiments in Pose validation, molecular preparation, and docking. YHu and XB performed the experiments *in vitro*. BY and JK discussed and reviewed the manuscript.

## Conflict of Interest Statement

The authors declare that the research was conducted in the absence of any commercial or financial relationships that could be construed as a potential conflict of interest.

## Supplementary Material

Figure S1 **(A)** The chemical structure of DT-13. **(B)** DT-13 showed no significant cytotoxicity on HUVECs at concentrations of 0.01–1 µM. **(C)** NMIIA was knocked down by a specific siRNA.

Figure S2 The abstract graph of this paper.
