# Supplementary figures and images for: DT-13 Ameliorates TNF-α-Induced Vascular Endothelial Hyperpermeability via Non-Muscle Myosin IIA and the Src/PI3K/Akt Signaling Pathway
Source: Front Immunol. 2017 Aug 14;8:925. doi: 10.3389/fimmu.2017.00925 (PMC5557769; doi:10.3389/fimmu.2017.00925)

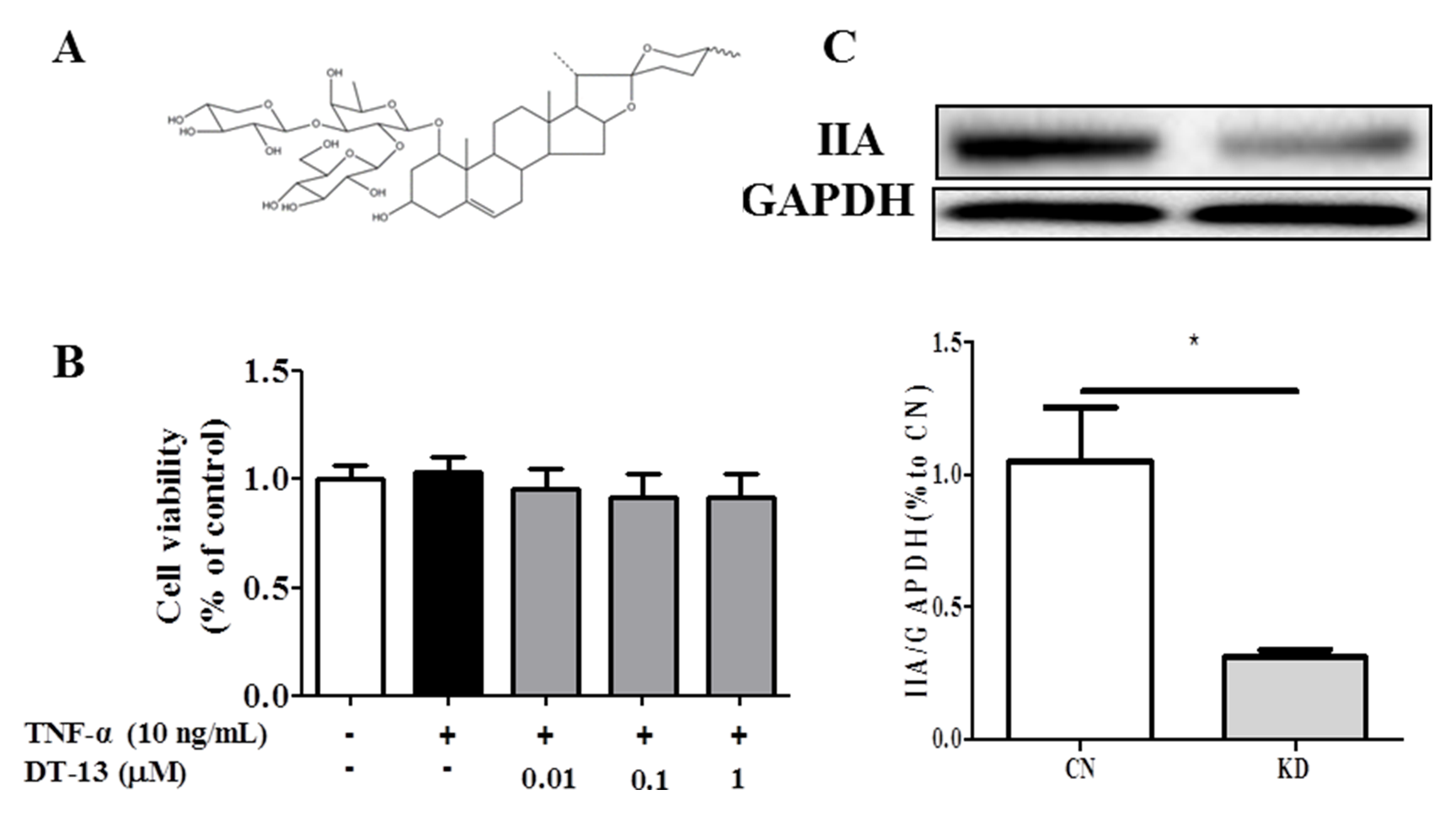

Supplement: Figure S1 — (A) The chemical structure of DT-13. (B) DT-13 showed no significant cytotoxicity on HUVECs at concentrations of 0.01–1 µM. (C) NMIIA was knocked down by a specific siRNA. [file Image_1.TIF]

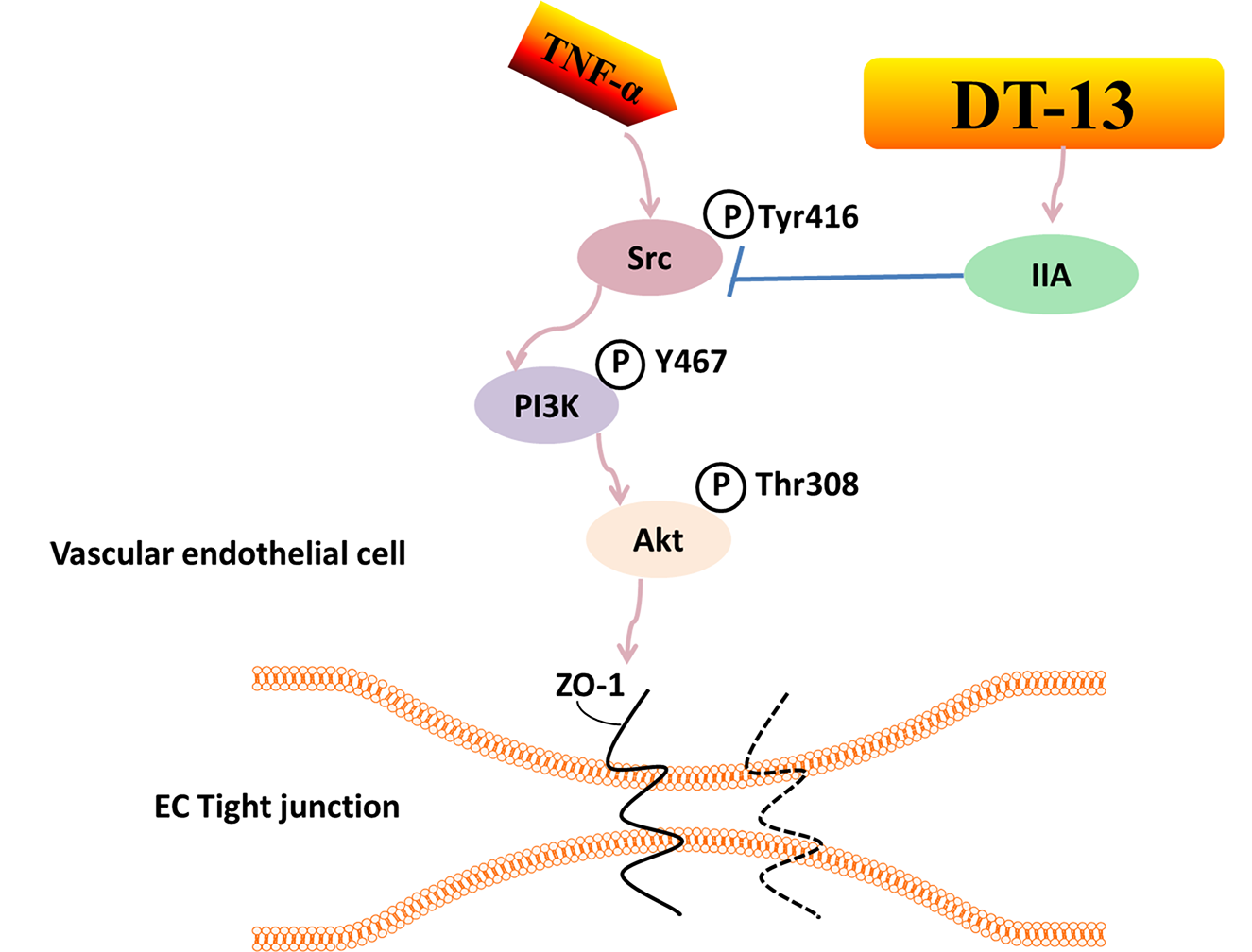

Supplement: Figure S2 — The abstract graph of this paper. [file Image_2.TIF]
